# An intervention mapping approach to the development and evaluation of a community-based prostate cancer early detection programme in Nigeria

**DOI:** 10.1371/journal.pgph.0004966

**Published:** 2025-08-05

**Authors:** Musliu Adetola Tolani, Christian Agbo Agbo, Shehu Salihu Umar, Alan Paciorek, Rufus Wale Ojewola, Faruk Mohammed, Mamoudou Maiga, Ernie Kaninjing, Muhammed Ahmed, Rebecca DeBoer

**Affiliations:** 1 Department of Surgery, Ahmadu Bello University/ Ahmadu Bello University Teaching Hospital, Shika-Zaria, Kaduna State, Nigeria; 2 Department of Surgery, Dalhatu Araf Specialist Hospital, Lafia, Nasarawa State, Nigeria; 3 Global Cancer Program, University of California, San Francisco, California, United States of America; 4 Department of Surgery, University of Lagos/ Lagos University Teaching Hospital, Idi-Araba, Lagos State, Nigeria; 5 Robert J. Havey, MD Institute for Global Health, Northwestern University Feinberg School of Medicine, Chicago, Illinois, United States of America; 6 School of Health and Human Performance, Georgia College and State University, Milledgeville, Georgia, United States of America; PLOS: Public Library of Science, UNITED STATES OF AMERICA

## Abstract

Interventions are needed to reduce the global disparity in prostate cancer outcomes. Our research aimed to evaluate the acceptability, feasibility and appropriateness of a stakeholder-developed intervention for the early detection of prostate cancer in Nigeria. This was a mixed methods study conducted at three cities in Nigeria. The intervention mapping framework was utilized as the primary framework for the study. During intervention development, a six-member team developed an implementation research logic model and then conducted stakeholder engagement to prioritize the preliminary intervention strategies among 12 purposively selected stakeholders. This was followed by brainstorming sessions by the same intervention development team for the selection of multi-faceted intervention strategies. Key informant interviews were then conducted among 10 purposively selected Nigerian clinicians, institutional leaders, and policymakers for contextual inquiry on the developed intervention. Descriptive statistics and qualitative analysis were performed. At the end of the intervention development process, the primary intervention strategies included supported risk assessment and patient navigation. The secondary strategies were education and training, and cancer information communication. The main actors of the intervention were trained community-based healthcare providers while the targeted population were adult men 40 years and above. The informants strongly noted that the intervention actions were feasible. Regarding acceptability, informants strongly believed that the intervention had numerous relative advantages but noted its complexity and the potential risk for overdiagnosis. Informants also consistently acknowledged that the intervention was appropriate but had mixed disposition on implementation preparedness. Further adaptations were made to address the stakeholder-identified gaps. In conclusion, this study developed a prostate cancer early detection intervention with preliminary feasibility, the first rigorously developed systems-strengthening intervention to optimize the detection of early-stage prostate cancer in Nigeria. A pilot implementation trial will be helpful in further testing the intervention in the field setting.

## Introduction

The mortality rate of prostate cancer in Nigeria (20.7 per 100,000) is around three-fold the rate in North America (8.3 per 100,000) and Western Europe (9.8 per 100000) [1]. Curative management for clinically localized prostate cancer (T1─T3a,N0,M0) presents an opportunity for intervention to reduce this poor survival outcome [2].

Using the principles of precision public health, dissemination and implementation science has been important in successfully building evidence to support tailored interventions for cancer management in Low- and Middle-Income Countries (LMICs) [3]. It has the potential to drive the optimal delivery of care to patients with clinically localized prostate cancer, and ultimately, to save lives, thereby, improving equity in global oncology [4]. Yet, the implementation of most interventions highlighted in the early cancer diagnosis recommendations of the World Health Organization (WHO), and their appraisal, are to a large extent, missing in the context of prostate cancer care in an LMIC like Nigeria [5,6]. This is because of the complex problems encountered during the detection of clinically localized prostate cancer as identified by multiple groups of stakeholders in Nigeria [7] These problems include variation in the skills and resources available to community-based healthcare providers and the relative centralization of access to early detection services in the country. Nevertheless, Nigeria’s cancer control plan prioritizes enhancing early cancer detection at the community level [8]. To support this goal and facilitate the swift translation of knowledge into practice, this study aimed to rigorously develop a holistic, patient-centered, and community-focused evidence-based systems strengthening intervention to target the complex problems encountered during the detection of clinically localized prostate cancer in Nigeria, and then conduct a contextual inquiry to evaluate its acceptability, feasibility and appropriateness.

## Materials and methods

### Study location

This study was conducted in Zaria, Lagos, and Lafia cities of Nigeria. The study locations were selected to ensure representation from three of the six geopolitical zones in Nigeria. The recruitment of human participants were conducted from 4^th^ July 2023–2^nd^ September 2023.

### Ethics statement

Ethical approval was obtained from the Health Research and Ethics Committee of Ahmadu Bello University Teaching Hospital, Zaria (ABUTHZ/HREC/F57/2021), Dalhatu Araf Specialist Hospital, Lafia (DASH/L/ADM/0166), and Lagos University Teaching Hospital, Lagos (ADM/DSCST/HREC/APP/4883) before the commencement of study activities. Written informed consent, was obtained from all study participants. Consent for the audio recording was also obtained from the key informants.

### Conceptual framework

A modification of the intervention mapping framework was employed to guide the development of the prostate cancer early detection programme [9,10]. This comprised of needs assessment, development of the logic model, selection of components for delivering the intervention that is matched with program objectives, and evaluation. Intervention mapping was favoured as these steps provided a basis for the integration of theory and evidence into major program planning activities [10]. The subsections below describe the application of the framework to this study.

### Intervention development

#### Needs assessment.

The process started with a situational analysis. This comprised of a cohort study on 110 patients with clinically localized prostate cancer and a qualitative exploration of barriers and facilitators of the detection of clinically localized prostate cancer among 52 stakeholders in Nigeria [7,11]. The cohort study demonstrated suboptimal quality of care including poor retention in care and geographical disparities in access to care [11]. The existing referral pathway for prostate cancer detection was also documented in this cohort study [11]. The qualitative study identified determinants of prostate cancer detection at the individual, interpersonal, and organizational levels [7]. The findings of these studies facilitated the identification of unmet needs and stakeholder-centred recommendations.

#### Development of logic model.

The intervention development team, comprising of four local investigators and two implementation science researchers, engaged in fortnightly hybrid meetings at Ahmadu Bello University Teaching Hospital, Zaria and online in order to design the logic model of the problem [12]. They mapped the identified determinants to the change that was earlier recommended by stakeholders and other evidence-based interventions in the literature [13]. This led to the generation of preliminary intervention strategies in four broad categories (cancer information communication, protocols, education and training, and environmental restructuring).

### Selection of intervention strategies.

#### Stakeholder engagement for intervention ranking.

Stakeholder engagement was conducted using the Tailored Implementation for Chronic Diseases’ (TICD) checklist for the prioritization of recommendations as the primary framework [14]. This checklist also recommends intervention ranking by at least two independent stakeholders. Prioritization generally depends on group dynamics rather than statistical power [15]. The intervention development team subsequently engaged 12 purposively sampled stakeholders in order to carefully select diverse participants who have adequate knowledge to adequately assess the intervention strategies. These stakeholders included seven implementation and dissemination associates (three doctors, two medical social welfare workers, one nurse, and one psychologist) and five targets of the intervention action (three patients as they were considered the primary target, and two caregivers). These stakeholders independently assessed three criteria (seriousness of the consequences of non-adherence, feasibility of the recommended practice in the targeted setting, and priority of implementing the recommendation) using a five-point scoring system (1 = No, 2 = Probably Not, 3 = Uncertain, 4 = Probably, 5 = Yes) and assigned a priority rank to the preliminary intervention strategies that formed the mapped intervention (S1 Data). Stakeholders also provided open-ended comments on the temporality and dose for the delivery of these intervention strategies. This process ensured that stakeholders in the health system of Nigeria had ownership of the intervention.

#### Brainstorming sessions for decision on the preliminary intervention strategies.

During brainstorming sessions, the intervention development team reflected on the intervention strategies using the Practical, Robust Implementation and Sustainability Model (PRISM) [16]. This model is based on the Chronic Care Model, the Model for Improvement, and the RE-AIM framework. It accounts for the identification of contextual factors as well as interactions between the proposed intervention, the recipients, the implementation and sustainability infrastructure, and the external environment. Given that this research looks at the delivery of prostate cancer care and treatment within hospitals in low-resource settings, PRISM is an excellent guide. The intervention development team discussed prioritized strategies with Likert scores of 4 or 5 and then divided the broad category of environmental restructuring into risk assessment and patient navigation. The intervention development team thereafter prioritized the strategies with the highest overall score in the categories of cancer information communication, protocols, education and training, risk assessment and patient navigation. More weight was given to the response of the end-users while reflecting on the overall importance of focusing efforts on each strategy as well as their relationship, sequence, and missing links with the top-ranking strategies.

### Intervention evaluation

#### Evaluation framework and study design.

Contextual inquiry on the developed prostate cancer early detection intervention was guided by Proctor’s taxonomy of implementation outcome [17]. It was based on a qualitative study design utilizing key informant interviews.

#### Sampling and recruitment.

A purposive sampling technique was used to select ten healthcare workers with diversity of expertise and experience in prostate cancer clinical management, institutional leadership, or national policy making [18]. We anticipated that at least ten key informant interviews will be adequate to achieve saturation, as there is little variation in information obtained after nine to ten interviews [19]. These participants were not a part of the needs assessment and prioritization phase of the project. The study team invited eligible individuals to take part in the interview, and those who agreed to participate were recruited.

#### Data collection.

The interviews were conducted in an environment where the privacy and confidentiality of the informants were assured. The facilitator (39 years old, male, urologist), who was a member of the intervention development team and has training in qualitative methods, presented the summary and conceptual diagram of the prostate cancer early detection intervention to the informants. Data was then collected using a face-validated interview guide to elicit information on the acceptability, feasibility, and appropriateness of the intervention [17] The interviews were audio recorded, transcribed and de-identified to maintain anonymity.

### Data analysis

The quantitative data obtained during the stakeholder engagement was analyzed using descriptive statistics with SPSS version 20 software (IBM Corp., Armonk, New York). Qualitative data analysis was done using the Framework technique, and its stages are transcription (by 28 years old, female, master’s degree qualification), familiarization, coding, analytical framework development, analytical framework application, data charting, and data interpretation [20]. A codebook was developed based on a priori concepts on the evaluation criteria from the interview guide and literature review. The interview transcripts were independently coded by at least two members of the research team with the Nvivo 12 software (QSR International Pty Ltd., Burlington, Massachusetts). Intercoder reliability was high with an average percentage agreement of 98.7%. We mapped the themes into the three evaluation criteria of acceptability, feasibility, and appropriateness. Sentiments were further categorized as negative, mixed, and positive tone of statements. The four pillars of trustworthiness (credibility, transferability, dependability, and confirmability) and reflexibility was addressed as detailed in Table 1. Acceptability, feasibility, and appropriateness were considered strong in strength if it was consistently coded positive in at least seven of the ten key informants.

**Table 1:** Trustworthiness and Reflexibility Strategies.

Pillars	Strategies
**Trustworthiness Strategies**	
**Credibility**	**Peer debriefing:** The research team regularly discussed to deeply examine emerging themes, cross-check interpretative decisions, and get alternative viewpoints.
	**Negative case analysis:** We systematically searched for salient interview responses that diverged from the predominant view of key informants, and the contradictory cases were further examined to refine our understanding of the implementation outcomes and guide further adaptation of the intervention.
	**Methodological Triangulation:** The study team corroborated the information obtained from the key informant interview with organizational documents (such as prostate cancer guidelines) in order to enhance the credibility of the interpretation of our findings and guide further adaptation of the intervention.
**Transferability**	**Thick descriptions:** The study team provided a description of the study participants, the study methodology, and the contexts and conditions for intervention implementation so as to allow readers understand the study setting and assess similarities or differences with other environment.
	**Purposive sampling:** This sampling strategy was employed to select key informants with different clinical roles and hierarchy, leadership positions, and policy-making status in order to obtain diverse views and expand the applicability of the study findings to similar populations outside the study context.
**Dependability**	**Audit Trail:** The study team maintained a record of the research procedures, including the interview guide, code book, and final analytic decisions and rationale. These ensured the transparency of the research process and findings. **Transcription checks:** This was done through the review of the transcript by a research team member to ensure that it is free from errors and omissions, therefore, increasing the trust on the captured data.
**Confirmability**	**Investigator triangulation:** At least two researchers independently coded each transcript and then discussed coding discrepancies in order to lower personal biases and strengthen consensus-based development of themes. **Confirmability Audit:** This was conducted by one qualitative researcher to ensure that interpretations were well supported by the study data. This minimized personal biases and strengthened the study’s objectivity.
**Reflexibility Strategies**	
**Research Team Positionality**	The research team included three urologists, one oncologist, and two qualitative research experts to enrich the understanding of the study context. All except one qualitative researcher has a previous clinical care role or hospital managerial experience within institutions similar to the study settings, and they provided initial insight into challenges that could be experienced in the study setting. All study team members, however, believe in the use of scientific data to close the knowledge gap. By explicitly acknowledging the perspective of the research team, readers can critically analyze the perspectives of the authors as it relates to this study.
**Reflexive Intervieweing Practices**	The research team actively managed positionality through asking open-ended questions to ensure that informants provided full, unprovoked responses; self-awareness of personal biases; and navigating possible power dynamics by creating a nonjudgmental atmosphere that encouraged candidness. These strategies ensured that the perspectives of the informants was central to the interview findings while also addressing personal and relational biases within the research team.

## Results

### Development of logic model

**Fig 1:**
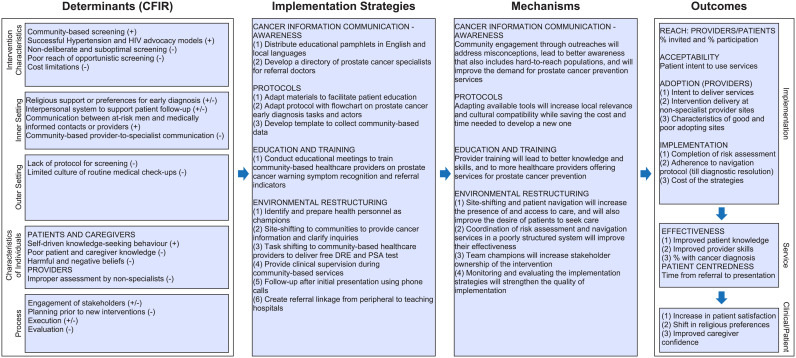
Logic Model for the prostate cancer early detection intervention. Abbreviations – DRE = Digital Rectal Examination, PSA = Prostate Specific Antigen. Logic model is based on the Implementation Research Logic Model Template of Smith et al [12].

The mapping of identified determinants to the recommended interventions led to the shortlist of 12 preliminary intervention strategies that were grouped into four broad categories based on their mechanisms of change in the determinants. The logic model of the intervention is presented in Fig 1.

### Stakeholder engagement for intervention ranking

Top-ranking strategies emerged during the prioritization of the 12 preliminary intervention strategies among the stakeholders. They included site shifting to communities, follow-up after initial presentation using phone calls, distribution of education messages, and conduct of educational meetings to train community-based healthcare providers. The priority rank for strategies in the category of protocols was the lowest (Table 2).

**Table 2:** Prioritization of recommended strategies by stakeholders.

S/No	Intervention Strategies	All Stakeholders^*^ Median (IQR)			Targeted Population Only* Median (IQR)		
		Are the consequences of non-adherence serious?	Is the suggested practice feasible in the targeted setting?	Is implementing the suggestion a priority?	Are the consequences of non-adherence serious?	Is the suggested practice feasible in the targeted setting?	Is implementing the suggestion a priority?
**Primary Intervention Strategies: Environmental Restructuring**							
A.	**Risk Assessment**						
1.	**Site-shifting to communities to provide cancer information and clarify inquiries**	4.0 (3.0 – 5.0)	4.0 (3.3 – 5.0)	4.5 (3.0 – 5.0)	4.0 (3.0 – 5.0)	4.0 (3.5 – 5.0)	5.0 (3.5 – 5.0)
2.	**Identify and prepare health personnel as champions**	4.5 (4.0 – 5.0)	4.0 (4.0 – 5.0)	4.0 (4.0 – 5.0)	5.0 (3.0 – 5.0)	4.0 (3.5 – 4.5)	4.0 (3.0 – 5.0)
3.	**Provide clinical supervision during community-based services**	4.0 (2.3 – 4.0)	4.0 (3.3 – 4.0)	4.0 (3.0 – 4.0)	3.0 (2.5 – 4.0)	4.0 (3.5 – 4.0)	4.0 (3.0 – 4.0)
4.	**Task shifting to CBHCPs (GMPs and CHPs) to deliver free DRE and PSA test**	4.0 (2.3 – 4.0)	4.0 (2.0 – 5.0)	4.0 (1.3 – 5.0)	4.0 (2.5 – 5.0)	3.0 (2.0 – 4.5)	4.0 (1.5 – 5.0)
B.	**Patient Navigation**						
1.	**Follow-up after initial presentation using phone calls**	5.0 (4.3 – 5.0)	5.0 (5.0 – 5.0)	5.0 (4.0 – 5.0)	5.0 (4.0 – 5.0)	5.0 (5.0 – 5.0)	5.0 (4.5 – 5.0)
2.	**Create referral linkage from peripheral to teaching hospitals**	5.0 (4.0 – 5.0)	4.5 (3.0 – 5.0)	4.0 (4.0 – 5.0)	5.0 (3.5 – 5.0)	4.0 (3.0 – 5.0)	4.0 (3.5 – 4.5)
**Secondary Intervention Strategies: Training And Communication**							
A.	**Cancer Information Communication/ Awareness**						
1.	**Distribute educational messages**	5.0 (5.0 – 5.0)	5.0 (5.0 – 5.0)	5.0 (5.0 – 5.0)	5.0 (5.0 – 5.0)	5.0 (4.0 – 5.0)	5.0 (5.0 – 5.0)
2.	**Develop a directory of prostate cancer specialists for referral doctors**	5.0 (4.3 – 5.0)	5.0 (5.0 – 5.0)	5.0 (5.0 – 5.0)	5.0 (2.5 – 5.0)	4.0 (3.5 – 4.5)	4.0 (3.5 – 5.0)
B.	**Protocols**						
1.	**Adapt materials to facilitate patient education**	5.0 (3.3 – 5.0)	4.0 (4.0 – 4.0)	5.0 (4.0 – 5.0)	4.0 (3.0 – 5.0)	4.0 (3.5 – 4.0)	4.0 (2.5 – 5.0)
2.	**Adapt protocol with flowchart on tasks and actors**	4.5 (3.0 – 5.0)	4.5 (3.0 – 5.0)	4.5 (3.3 – 5.0)	3.0 (2.5 – 5.0)	3.0 (3.0 – 5.0)	3.0 (3.0 – 4.5)
3.	**Develop template to collect community-based data**	4.0 (3.0 – 4.0)	4.0 (3.3 – 5.0)	4.0 (3.0 – 4.0)	4.0 (3.0 – 4.5)	4.0 (2.5 – 4.5)	4.0 (2.5 – 4.5)
C.	**Education And Training**						
1.	**Conduct educational meetings to train CBHCPs (GMPs and CHPs) on prostate cancer warning symptom recognition and referral indicators**	5.0 (4.0 – 5.0)	4.5 (3.3 – 5.0)	5.0 (4.0 – 5.0)	5.0 (3.5 – 5.0)	4.0 (3.0 – 5.0)	5.0 (3.5 – 5.0)

Abbreviations – CBHCPs = Community-based Healthcare Provider, CHP = Community Healthcare Practitioner, DRE = Digital Rectal Examination, GMP = General Medical Practitioner, IQR = Interquartile Range, PSA = Prostate Specific Antigen.

*A 5-point Likert Scale (1 = No, 2 = Probably Not, 3 = Uncertain, 4 = Probably, 5 = Yes) was used for the prioritization process. A lower score indicates that its priority is likely to be low.

### Brainstorming sessions for decision on the preliminary intervention strategies

At the end of the brainstorming process on the prioritized interventions, the intervention development team selected composite strategies comprising primary and secondary intervention packages. The primary intervention package included supported risk assessment and patient navigation that will be delivered by trained community-based healthcare providers (general medical practitioners and community healthcare practitioners) as the main actors. The intervention will target adult men 40 years and above (Table 3). The secondary intervention package included education and training that will be delivered by oncology specialists to community-based healthcare providers as well as cancer information communication/awareness that will be delivered by community-based healthcare providers to adult men 40 years and above, their family members, and community and religious leaders (Table 4). The suggestions of the stakeholders on the dose and temporality as well as the RE-AIM framework were further considered during the intervention development phase to develop the building blocks of the primary and secondary intervention packages.

**Table 3:** A summary of the prostate cancer early detection primary intervention package on supported risk assessment and patient navigation.

Building Blocks	Components
**Actors**	• **Community-based healthcare providers** • Community healthcare practitioners • General medical practitioners
**Targets of actions**	• **Clinical characteristics of targets:** Adult men 40 years and above
**Actions, Temporality and Dose**	• Supported risk assessment – Supervised site-shifted cancer risk assessment in primary healthcare centres and general hospitals by digital rectal examination and prostate specific antigen test, and cancer information and risk clarification – *Once monthly* • Patient Navigation – Referral linkage and follow-up (phone call) – *After access to initial cancer service*
**Implementation Outcome:** **short term**	**Short term: Supported Risk Assessment** • Reach/ Penetration: Proportion of patients agreeing to participate in risk assessment • Adoption: Proportion of providers who deliver the services and characteristics of good and poorly performing sites • Implementation: • Completion of risk assessment tasks by providers • Ease of provider understanding of risk assessment tasks • Actual fit • Suitability for risk assessment for everyday use • Acceptability: • Relevance and relative advantage of the risk assessment • Complexity of risk assessment tasks • Effectiveness (exploratory): Proportion of patients with risk assessment who were diagnosed with prostate cancer. **Short term: Patient Navigation** • Adoption: Proportion of providers who deliver the services and characteristics of good and poorly performing sites • Implementation: Proportion of patients with referral in accordance with the intervention protocol (follow-up of patients until diagnostic resolution)
**Implementation Outcome:** **long term**	**Long-term: Supported Risk Assessment** • Effectiveness: Proportion of those with risk assessment who were diagnosed with prostate cancer • Satisfaction: Proportion of patients satisfied with risk assessment • Implementation cost: Cost per unit of early cancer diagnosis **Long-term: Patient Navigation** • Effectiveness: Proportion of patients with successful navigation • Timeliness: Time from referral to presentation to specialists • Satisfaction: Proportion of patients satisfied with patient navigation • Implementation cost: Cost per unit of early cancer diagnosis
**Justification**	PRISMA Framework

**Table 4:** A summary of the prostate cancer early detection secondary intervention package on education and training plus cancer information communication.

Building Blocks	Components
**Actors**	• **Oncology specialists (Education and Training):** Urologists, Oncologists • **Community-based healthcare providers (Cancer Information Communication)**
**Targets of actions**	• **Socio-demographic characteristic of education and training target participants**: Community-based healthcare provider actors (community healthcare practitioners and general medical practitioners) working in primary healthcare centers and general hospitals • **Characteristics of cancer information communication targets:** Adult men 40 years and above, their family members, and community and religious leaders
**Actions, Temporality and Dose**	**Education and Training** • Conduct educational meetings to train participants on prostate cancer warning symptom recognition and referral indicators – *One usual working hour, twice only at 3-month intervals* **Cancer Information Communication** • Adapt materials to facilitate patient education – *At the end of the second refresher educational meeting* • Conduct health talk and distribute educational messages during outreach visits (churches, mosques, townhalls, rural communities) – *30 minutes talk and 30 minutes feedback, once only per cluster*
**Implementation Outcome**	**Education and Training** • Reach: Proportion of nominated participants who attend the training • Adoption: Proportion of providers who intend to deliver the service • Effectiveness: Improved provider knowledge and skills on prostate cancer **Cancer Information Communication** • Reach/ Penetration: • Number of participants or men ≥ 40 years accessing health talk • Number of participants with intent to use the service • Timeliness: Time from health talk to hospital presentation for assessment • Effectiveness: Improved patient knowledge and understanding of prostate cancer • Change in attitude: Shift in religious opinion towards the need for early hospital testing over traditional healing
**Justification**	PRISMA Framework

### Intervention evaluation

A total of ten Key Informant Interviews (KII) were conducted. A summary of the intervention strategies is presented on Tables 3 and 4. Their demographic details of the participants are summarized on Table 5. The mean duration of the interviews was 37 minutes (range, 31 – 53 minutes). A total of 553 items were coded.

**Table 5:** Demographic detail of the key informants (n = 10).

Characteristics	Values*
**Age (years)**	46 (36 – 55)
**Gender**	
Female	2 (20)
Male	8 (80)
**Geographical zone**	
North West	4 (40)
North Central	3 (30)
South West	3 (30)
**Role**	
Urologist	1 (10)
Clinical oncologist	1 (10)
Radiation oncologist	1 (10)
Radiologist	1(10)
Nurse	2 (20)
Community healthcare practitioner	1 (10)
Institutional leader	2 (20)
National health policy maker	1 (10)
**Professional Experience, (years)**	14 (3 – 20)

*All values are expressed as frequency (percentage) except for age and professional experience which was expressed as median (range).

#### Acceptability.

##### Relative advantage.

Almost all of the participants made overwhelmingly positive comments on the advantage of this tailored early detection programme compared to what was obtainable in prostate cancer programmes in Nigeria. They believed that the intervention was a radical change and active process that will help increase knowledge of prostate cancer among community-based healthcare providers; make the general public more conscious about prostate cancer; and afford adult males at risk better chance for disease detection and cure. A radiologist further noted: *“It seems to be all encompassing considering the fact that it involves even those at the primary healthcare level that are closer to these patients”* (Quote #1) and explained that this will boost their confidence to seek care. Another important dimension highlighted was the potential of the programme to have broader reach to people and communities beyond the coverage of the routine services on prostate cancer (Quote #2). One of the institutional leaders however emphasized more on relative shortcomings. This included non-integration into routine care as a potential setback for the sustainability of the programme (Quote #3). He also cited the drawback of overdiagnosis stating that: *“Ultimately, any intervention like this that actively seeks to detect prostate cancer early has to also proactively think about the ultimate consequences of overdiagnosis”* (Quote #4).

##### Complexity.

While evaluating potential difficulties of various components of the intervention, the opinion of majority of the participants strongly tilted towards a perception of high complexity of the strategies. They strongly cited the traditional pathway of contact with the target population which requires the buy-in of religious and community leaders (Quote #5), uncertainty about the duration of time needed to adequately train the actors prior to conducting the shifted task (Quote #6), variation in the information needs of clients in the rural and urban communities (Quote #7) and the poor culture of conducting follow-ups in this setting (Quote #8). Despite these challenges, three of the participants however strongly argued to support the ease of conducting the program. One of the them, a community-based nurse, saliently mentioned that: *“I think that it is a straight forward program. I think the scope is great and encompassing enough for an early start”* (Quote #9).

##### Satisfaction.

The comments of all the informants about their pleasure with the comfort or the delivery channel of the programme was moderate in strength and mixed in tone. One of them, a radiation oncologist, stated that the intervention was timely and valuable (Quote #10) and another one, an oncology nurse, was particularly pleased with the selection of primary heathcare centres as the delivery channel (Quote #11). On the other hand, a clinical oncologist noted the lack of details into how the targeted patients will be able to access specialist care (Quote #12) as the reason for his dissatisfaction (Table 6).

**Table 6:** Representative quotes reflecting pre-implementation acceptability of the prostate cancer early detection intervention in Nigeria.

Evaluation Domain	Representative quotes
**Relative advantage**	#1 “It seems to be all encompassing considering the fact that it involves even those at the primary healthcare level that are closer to these patients. And for some of them, they have relations (in the community). So, by involving them in early diagnosis, I think that it will go a long way in even boosting the confidence of these patients” (Radiologist, 38 years old)
	#2 “More people will be diagnosed and a wider community of people will be reached if this is implemented. That means that they have a better shot at being cured compared to coming at the late stage” (Community-based nurse, 33 years old)
	#3 “So, sustainability is an issue one has to talk about because these are activities that are done outside routine (practice) and as long as there is no form of reward or remuneration attached to such practices, they ultimately fade away, in the event that doing it or not doing it has no consequences” (National professional association leader, 50 years old)
	#4 “Ultimately, any intervention like this that actively seeks to detect prostate cancer early has to also proactively think about the ultimate consequences of overdiagnosis” (National professional association leader, 50 years old)
**Complexity**	#5 “(We should implement the program) in such a way that the religious and community leaders will be involved. Every community has their setting, cultural beliefs and how they confront members of the community. So, bringing the community leaders and the religious leaders into play first even before starting is very important” (Oncology nurse, 39 years old)
	#6 “Well, if I should look at the content needed for this programme, the time may not be enough. You have to determine how long you want to train them before you think you can release them, because it is so important that they are trained well” (Urologist, 55 years old)
	#7 Assuming we have two scenarios, somebody from a semi urban or urban area and then another from a rural area, the level of feedback and questions that will be asked and the explanation on what you intend to do will be more for the rural participants than for the urban participants” (National policy maker, 47 years old)
	#8 “The communication culture around here is really poor. And it is seldom that you find someone making follow-ups on initial task they have set out to do” (Radiation Oncologist, 40 years old)
	#9 “I think that it is a straight forward program. I think the scope is great and encompassing enough for an early start” (Community-based nurse, 33 years old)
**Satisfaction**	#10 “Well, first I think the intervention is timely and it is valuable” (Radiation Oncologist, 40 years old)
	#11 “All the channels are the best location where you can get people to listen and understand what is going on. Involving primary healthcare is far much better than sending them to a bigger setting of hospital directly for screening and all these assessments. When you use the location, it is easier for them because many people are used to going there for questions and health interventions” (Oncology nurse, 39 years old)
	#12 “Can they access a specialist to get the best intervention in terms of treatment? Basically, these are my concerns for this program” (Clinical oncologist, 37 years old)

#### Feasibility.

##### Actual fit.

All the informants strongly noted that the actions of the intervention will be easy to understand by the targeted population. They explained that the presence of training as a part of the intervention will make the community-based healthcare providers comprehend the actions in the programme (Quote #13). A community healthcare practitioner and nurse further referenced that the understanding of the general population will be easier with the use of locally based actors, who speak their language dialect or pidgin English (Quote #14) and who are the most likely people that can convince them to seek care (Quote #15).

##### Suitability.

Majority of the informants believed that it will be easy for the actors delivering the interventions to carry out the assigned tasks. One of them explained that the baseline experience of the proposed actors in the delivery of other community-based health programmes will facilitate the intervention (Quote #16). A few of them, however weakly opined that it was not going to be easy. They referenced lack of prerequisite knowledge of prostate cancer among actors (Quote #17), potential rivalry arising from unclear assignment of roles for the health education and risk assessment tasks (Quote #18), and possible demotivation arising from the lack of community ownership (Quote #19).

##### Trialability.

Almost all informants proposed that a pilot study was necessary to assess this programme. They explained that it will test the practicability of the intervention (Quote #20) and will help to generate more interest in the community about the programme (Quote #21). However, the health institution leader, who had a mixed opinion, believed that the intervention was good enough to be implemented on full scale with a nested pilot phase in order to save cost (Quote #22) (Table 7).

**Table 7:** Representative quotes reflecting pre-implementation feasibility of the prostate cancer early detection intervention in Nigeria.

Evaluation Domain	Representative quotes
**Actual fit**	#13 “I think that these actions will actually work well in our community when doctors in primary health care center, nurses, and the community healthcare practitioners are actually trained to deliver the health education and assessment activities” (Clinical oncologist, 37 years old)
	#14 “The people who will be reaching out to these patients are the healthcare workers in the community who live with them. They will be able to speak in their language and interact with them. So, I think yes, it will be easy for the clients to understand the actions. It does not even require them to do much other than for them to just be present, and hear what they want to say for them to understand” (Community-based nurse, 33 years old)
	#15 “They are the ones that can convince the patients. Believe me, sincerely, that there are some patients that will not answer the doctor if the community healthcare practitioner does not tell them that it is beyond them” (Community healthcare practitioner, 37 years old)
**Suitability**	#16 “Yes, I believe that it will be easy because these healthcare workers are not new to deploying programs such as these. We can see that they deploy programs such as immunization, tuberculosis management program and all that” (Community-based nurse, 33 years old)
	#17 “It is not going to be easy because they don’t have the prerequisite knowledge of prostate cancer. Normally, even some graduate doctors don’t have this prerequisite knowledge” (Health institution leader, 58 years old)
	#18 “The problem may now (be) how to deal with the personal issues of hierarchy or seniority (across healthcare professions). So, it may now be personal interests that may be different and be a challenge in terms of ‘Who does what?’, ‘I can do it’ and all that” (Clinical oncologist, 37 years old)
	#19 “People at the primary healthcare level will buy more into this if they see it as ‘our own thing’. You will see that, immunization for instance, is being embraced at the primary healthcare level because they see it majorly as their own. With this, we would have people that are motivated to carry out this course” (Community-based nurse, 33 years old)
**Trialability**	#20 “I think that there are some aspects of this intervention that may need alteration but because we have not test run it, we may not know. So, I believe from test running, we will get to know those aspects (requiring modification) that were not obvious to us” (Radiologist, 38 years old)
	#21 “Starting from a specific area will help. (For example), from a small community, your first contact will deliver the message to many people. So, (with) people having the information before you go there, I think (that it) will have (a) better impact” (Oncology nurse, 39 years old)
	#22 “It will be helpful quite a lot because you will use that to assess the response of people within the community whether they are responding positively or not. You can use that to assess the response of a community health worker to your education and their cooperation too. Your training will assist in giving information, health talks and all that… Well, I am looking at the cost. If you want to pilot this intervention program, that means the cost will be double, and then where are you going to get the resources? You can carry on (the intervention) and make (the pilot) as part (of the intervention) to assess yourself and (then) continue” (Health institutional leader, 58 years old)

#### Appropriateness.

##### Compatibility.

Compatibility was the most coded item in the interview. All the informants consistently acknowledged that the programme was compatible with their individual norms. A clinical oncologist referenced that it aligned with his perception of making a difference in the outcome of patients (Quote #23) while a urologist stated that: *“I, as a consultant, want to have patients with early diagnosis. We want patients that you can at least give a hope of cure rather than patients whom you know that the only thing you can do is just palliation”* (Quote #24). All the informants also reiterated that the interventions align with their organizational goals and matches with the existing national policy on cancer control. The national policy maker summarized: *“So, any program that is targeted on prevention, definitely fits into national preventive programs on cancer, either at the primary, secondary or tertiary levels”* (Quote #25). Almost all the Informants also noted that it will complement the existing work process as it will boost the existing surveillance and referral system at the primary healthcare level (Quote #26). Majority also felt that the intervention strategies was well suited with the literacy level of the actors (Quote #27). However, two other informants, the health institutional leader and the urologist, saliently noted that the knowledge of community-based healthcare providers on prostate cancer is limited (Quote #28) and their competency to adequately deliver treatment-related information without misleading patients is doubtful (Quote #29).

##### Tension for change.

Although the opinions of the informants were weak in tone, majority of them cited the involvement of religious leaders (Quote #30), and of community participation through community-based healthcare providers as strategies that makes the intervention culturally responsive and facilitate its integration into care (Quote #31). The intervention by design could also address cultural issues related to testicular loss. One of the participants noted: *“When a man is told that he has to undergo bilateral orchiectomy, that is when they begin to bring issues of culture. So, I think from that perspective, it offered a way forward they should sit well with the clients”* (Quote #32). The only informant, who expressed reservation on the cultural responsiveness of the intervention, opined that decision during the illness of a individual is usually collectively taken by family members (Quote #33)

Five of the ten informants also spoke in moderate tones about the relevance of the intervention in reorganizing early cancer detection (Quote #34), and reducing the resources needed to cater for these patients in the long run (Quote #35).

##### Readiness for implementation.

There were mixed coding as regards the availability of resources for the implementation of this intervention**.** Some of the informants highlighted the ready availability of allied healthcare workers (Quote #36), dedicated space within the facility, and the potential for donor agency funding (Quote #37). In contrast to this, some of the other respondents, expressed concerns on the availability of adequate funding for the intervention activities (Quote #38). Nevertheless, A national professional association leader balanced these views stating that: *“I will rather think (that) this will serve as a foundation or a bedrock on which further improvements can be built upon. It is almost like laying an initial (foundation) for what wasn’t there before. Or we just used practices that were not coordinated. Now, you are bringing it all together in a structured manner, (in order) to better focus with clear-cut outcomes”* (Quote #39) (Table 8).

**Table 8:** Representative quotes reflecting pre-implementation appropriateness of the prostate cancer early detection programme in Nigeria.

Evaluation Domain	Representative quotes
**Compatibility**	#23 “It aligns with my own perception of making a difference in improving patients’ outcome” (Clinical oncologist, 37 years old)
	#24 L04: “I, as a consultant, want to have patients with early diagnosis. We want patients that you can at least give a hope of cure rather than patients whom you know that the only thing you can do is just palliation” (Urologist, 55 years old)
	#25 “So, any program that is targeted on prevention, definitely fits into national preventive programs on cancer, either at the primary, secondary or tertiary levels” (National health policy maker, 47 years old)
	#26 “It will complement (the existing process) because this (intervention) is adding value to the surveillance system in our health facility. So, once you bring it in, ……., you are good to go, there is no issue” (Community healthcare practitioner, 37 years old)
	#27 “I think the fact that the community healthcare practitioners, the nurses, and the doctors in my setting are actually equipped to educate these patients and persuade them to take charge (of the process)” (Clinical oncologist, 37 years old)
	#28 “Because they don’t have that concise knowledge of prostate cancer in terms of training, so it is not going to be easy with them” (Health institutional leader, 58 years old)
	#29 “The treatment options for prostate cancer are numerous and I am not sure whether they will be able to deliver this information adequately without patients being misled” (Urologist, 55 years old)
**Tension for change**	#30 “In the intervention, there is an aspect where religious leaders and people at the religious centers will also have some form of health talks conducted in such places. That, to a large extent, can soften the ground and make it easier for people to accept to proceed for further evaluation” (Urologist, 55 years old)
	#31 “And then, community participation through the community healthcare practitioners or the primary healthcare workers around them, that are known to them. I think that would make it a bit more culturally accepted to the targeted participants. At the same time the intervention will be a bit more relevant and can easily get integrated” (National health policy maker, 47 years old)
	#32 “When a man is told that he has to undergo bilateral orchiectomy, that is when they begin to bring issues of culture. So, I think from that perspective, it offered a way forward they should sit well with the clients” (Radiologist, 38 years old)
	#33 “Cultural peculiarities are mostly related to family size, multiplicity of wives, extended family setting, and so on. Even taking decisions on one’s own health often does not just lie in the hands of the person who is sick. It is often a family decision and one of the aspects that affect that is the cost of health care. And that of course affect the ultimate outcome” (National professional association leader, 50 years old)
	#34 “So, there is a significant need to reorganize the system. Perhaps the outcome of this study might advise the management to have a very clear-cut organization on cancer detections generally not just prostate cancer and have a clear work plan on how to achieve that” (National professional association leader, 50 years old)
	#35 “So, once more patients are diagnose early, in the long run, the resources that is expended by government, by the hospital management to cater to prostate cancer patient will ultimately be reduced” (Radiation Oncologist, 40 years old)
**Readiness for implementation**	#36 “I also think that we have the human resources that will be necessary to see to the success of the intervention. Because we have community healthcare practitioners, nurses, and physicians that work in almost every locality in the area where this intervention is been plan to be deployed” (Radiation Oncologist, 40 years old)
	#37 “So, we can get one of these to partner with NGOs that are interested in prostate cancer, and people in government that can help us table these at the top, to top government officials. I think we will be able to get much funding to do it” (Community-based nurse, 33 years old)
	#38 “So, our intervention is going to be affected by healthcare financing and in fact out-of-pocket payment…. The aspect of the intervention perhaps which, maybe, needs to be addressed is that of financing. Who pays for the PSA test? If the patient cannot afford it despite the fact that he needs it, how do you go about it? Health insurance does not cover for it. Can there be other ways to cover for that?” (National professional association leader, 50 years old)
	#39 “I will rather think (that) this will serve as a foundation or a bedrock on which further improvements can be built upon. It is almost like laying an initial (foundation) for what wasn’t there before. Or we just used practices that were not coordinated. Now, you are bringing it all together in a structured manner, (in order) to better focus with clear-cut outcomes” (National professional association leader, 50 years old)

#### Further adaptations for intervention refinement

**Fig 2:**
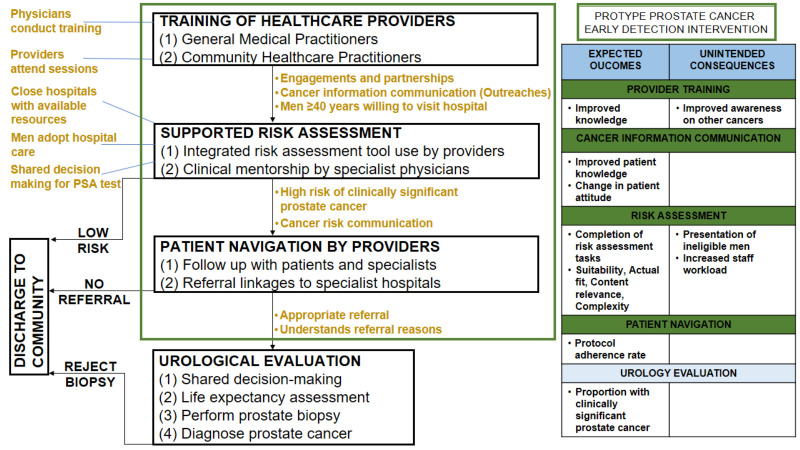
Conceptual diagram for the prostate cancer early detection intervention. Gold-coloured fonts: conditions for intervention success.

The intervention strategies were further modified using stakeholder feedback obtained from the contextual inquiry. Adaptations to address implementation readiness and complexity included the addition of conditions for intervention success such as financial and other material resource needs, and engagements and partnerships with providers, the community, and donors. The intensity of provider training was also modified to half-day training for a consecutive period of three days. The refresher courses for providers were substituted and the initially proposed supervision intensified through weekly clinical mentorship and activity checklist review during the supported risk assessment. The clinical mentors will be specialists with at least three years of experience in prostate cancer management. Furthermore, the use of risk assessment tools (such as integrated clinical, biochemical, or imaging markers or their combination) to optimize the diagnosis of clinically significant prostate cancer was added to address the salient point on the risk of overdiagnosis despite the relative advantage of the intervention. In line with this addition, cancer information communication was expanded to include both community outreaches for general awareness and peri-risk assessment communication for information provision on individual cancer risk. In addition to the patient navigation which remained unchanged because the intervention development team anticipated that it will address the poor medical follow-up culture, the elements of urological evaluation for prostate cancer diagnosis were specified in order to highlight how the targeted patients will be able to access specialist care. Positive and negative unintended consequences of the intervention were also highlighted. The inter-relationship of the intervention strategies are shown in Fig 2.

Healthcare provider training will be conducted by specialist physicians. Through engagement and partnerships between community-level hospitals and tertiary healthcare centres, the trained healthcare providers will conduct outreaches and navigate men 40 years and above to community-level hospitals for supported risk assessment. The trained healthcare providers will evaluate the disease risk of participants using an integrated risk assessment tool under the clinical mentorship of specialist physicians. All evaluated men will be counselled about their risk of developing the disease. For men predicted to have a high risk of clinically significant cancer, they will be navigated to a urology specialist. The patient navigation will involve trained healthcare providers facilitating referral linkages to the appropriate specialist hospital, and contacting patients and urologists for follow-up on completing a clinical appointment. For participants understanding the importance of referral, they will present for urological evaluation and undergo shared decision making, life expectancy assessment, biopsy performance, and the diagnosis of prostate cancer.

## Discussion

This study innovatively used mixed methods and an intervention mapping framework to rigorously develop and evaluate the prostate cancer early detection intervention for the context of patients at risk of prostate cancer in Nigeria. The overall result showed that the developed tool had preliminary feasibility. Further adaptations were made to develop a pilot-ready intervention for the optimization of prostate cancer early detection in the Nigerian setting.

The multifaceted, stakeholder-prioritized, prostate cancer early detection intervention, evaluated in this study represents a contextually relevant intervention to improve the quality of care, cause a downward shift in the stage at diagnosis of prostate cancer, and tilt its detection to predominantly curable disease in a resource-limited setting. The finding in this study which cited the salient concern of overdiagnosis is a common rationale for the limited attention to the development of routine programmes for the early detection of prostate cancer in the Nigerian setting. Kensler et al. have identified age, race, and family history as strong risk factors for prostate cancer [21] Song et al. also documented that the rate of moderate to severe lower urinary tract symptoms in patients with newly diagnosed localized prostate cancer is 62.9% [22] The evaluated model in this study, therefore, incorporated a risk-based approach of susceptibility clarification and symptom assessment before prostate specific antigen testing as part of the environmental restructuring needed to balance the concerns of overdiagnosis while maximizing the yield of diagnosis in the patients and efficiency of resource utilization in the health system [23] This pathway aligns with the proposal of Sarma et al. on the need to focus efforts on the early detection of symptomatic cancers to reduce missed opportunities [24] Mandal et al. further noted that the resulting overtreatment could be mitigated by the use of a practical protocol of active surveillance in patients with low risk diseases [25]

The perception of potential relative advantages of improved community and provider knowledge, better reach of routine services for prostate cancer, more chance of detection of clinically significant prostate cancer in its earliest stage, and its comparison to the present non-existence of routine community-based prostate cancer early detection intervention are indicators of the strong acceptability of this programme. A trigger-based intervention has been used to improve the timeliness of evaluation of features suggestive of prostate cancer [26] Unlike their study where the trigger had some level of automation from electronic records, the trigger for prompt referral will be activated by community-based healthcare providers in this study in line with the resource limitations in Nigeria. The finding in this study strongly acknowledged the high complexity of our community-based approach in terms of the uncertain duration for the training of the community-based healthcare providers and addressing information needs in rural areas. Despite its considerable acceptability, Taylor et al. also highlighted the extra burden related to the additional time and engagement needed during population-based cancer detection programmes [23] Similar to this study, the majority of the retrieved articles in this systematic review were qualitative in study design and the studies were based on the perspectives of healthcare providers. However, the extracted data were related to the context of breast and ovarian cancer detection in five high-income countries. Despite the difference, these findings have important implications during the refinement, pilot or scale-up of our intervention in order to enhance its acceptability.

Findings from this study also reflected satisfaction by some informants regarding the selection of community-based hospitals, such as primary healthcare centres, as a delivery channel for the intervention. This could be explained by the perceived reduction in the barriers of accessing prostate cancer risk assessment services by men at risk of the disease. This satisfaction could also reflect provider-level consequence of the developed intervention as Ibekwe et al. observed that their training intervention improved the skills, attitude and confidence of providers and navigators towards cancer screening and early detection [27] Nevertheless, the negative view of another informant, which was in contrast with this positive opinion, highlights the strong need for the development of an intervention which completes the entire pathway of diagnosis of prostate cancer in those at risk of the disease. As a result, the intervention was further refined to include a pathway to urological evaluation so as to clearly map how the patients will be able to access specialist care.

The references made to the use of trained health workers with prior involvement in community-based programmes and familiarity with the setting as an illustration of the feasibility and cultural responsiveness of the intervention services in our programme reflects the contextual significance attached to the use of a community-engaged approach to secure the buy-in of the target population. The positive perception of informants on the inclusion of religious leaders in the awareness component of our intervention means that they present an important space for social interaction. Similar to this study, Benedict et al. in South Africa identified sites for community gatherings like religious meetings as important in building a strategy for educating the public on prostate cancer risk factors so as to enhance its detection [28] However, these strategies were developed through a literature review and the Delphi process, unlike our study that utilized a framework to map known determinants to actions needed to effect change. Fu et al. noted that the use of historically safe social and community spaces that empower its people can build public trust in the process and improve the reach of the programme [29] Informants however noted that a pilot study will add more value to the final developed plan. This aligns with Johnson et al.’s perspective that a stepwise approach is needed during the implementation of innovation in various aspects of cancer control in order to generate field-based data on the determinants of adoption, iteratively address these challenges, and achieve incremental success in universal health coverage [30].

The strong perception of all the ten informants, with diverse provider roles, on the compatibility of the programme with their norms of giving patients hope of care, organizational goals of supporting prevention, and existing work process of community surveillance, means that they consider it appropriate to implement. This therefore provides an open opportunity to strengthen the impact of this intervention on the public health integration, clinical management, and national cancer control policy front. Moreover, definitive treatment for prostate cancer, through radical prostatectomy or radiotherapy, can be performed across tertiary hospitals in Nigeria, therefore, providing a chance to leverage these services to offer curative treatment after the completion of diagnostic workup [31–33]. Despite these strengths, informants acknowledged funding constraints as a major impediment to the success and sustainability of the programme. Notwithstanding, men in the community setting in Nigeria have a high demand for and are willing to pay a fraction of the cost during the implementation of a population-based programme for the early detection of prostate cancer [34]. On one hand, this indicates the need for innovative financing options to support this service at the primary care level. On the other hand, there appears to be a need to model the dynamics of resources including cost and other complexities while piloting or refining this intervention to produce a sustainable programme.

## Conclusion

Overall, the prostate cancer early detection intervention had preliminary feasibility. Further adaptations was done based on the stakeholder feedback to address potential challenges of implementation readiness, complexity, and overdiagnosis. A pilot implementation trial will be helpful to further test the refined intervention in the field setting.

## Supporting information

S1 Data Intervention ranking by stakeholders. (XLSX)
